# Effects of immunomodulating therapies on mortality in patients with severe cutaneous adverse reactions in comparison with supportive care only: a systematic review

**DOI:** 10.1186/2045-7022-4-S3-P15

**Published:** 2014-07-18

**Authors:** Stefanie Zimmermann, Peggy Sekula, Moritz Venhoff, Jean Claude Roujeau, Martin Schumacher, Maja Mockenhaupt

**Affiliations:** 1 Dokumentationszentrum schwerer Hautreaktionen (dZh), Department of Dermatology, Medical Center, University of Freiburg, Germany; 2 Center for Medical Biometry and Medical Informatics, Medical Center, University of Freiburg, Germany; 3 Department of Dermatology, Reference Center for Toxic and Autoimmune Blistering Disease, Hôpital Henri Mondor, University Paris-Est Créteil, France

## Background

Stevens-Johnson syndrome and toxic epidermal necrolysis (SJS/TEN) are severe cutaneous adverse reactions that are associated with high mortality. Mainly due to rareness, but also due to unpredictable onset and rapid course, therapeutic effects are often studied in observational settings. Since an evidence-based standardized treatment protocol for SJS/TEN is still lacking, we intended to combine information and evidence from any type of study to provide a comprehensive overview on immunomodulating therapies and their potential effects in comparison to supportive care only.

## Methods

A systematic literature search regarding publications on therapy of SJS/TEN was performed for the period from 1990 to 2012. Any type of study meeting predefined inclusion criteria was included. Data were extracted from published articles. Study groups were contacted to get further information. Eventually, all studies were assessed for final inclusion. To evaluate therapeutic effects we conducted a meta-analyses based on aggregated as well as on individual patient data (IPD).

## Results

Besides dozens of observational studies only one randomized controlled trial have been found. The quality of most studies has been assessed as low. 96 studies including 3451 patients passed our selection process. The majority of them focussed on SJS/TEN-patients treated with supportive care, glucocorticosteroids or intravenous immunoglobulines. Few studies evaluated other immunomodulating therapies like ciclosporin, plasmapheresis, cyclophosphamide and thalidomide. Besides monotherapy with one immunomodulating agent combined treatment modalities were also observed. Only 17 studies (18%) assessed therapy effects and are thus principally eligible for inclusion in our common meta-analysis. For the IPD analysis, 1209 patients (35%) with complete information are available. Both analyses are currently ongoing. The results will be presented.

## Conclusion

The comprehensive overview about possible therapies for SJS/TEN revealed no unexpected treatment options. Final results of both analyses are awaited for estimating the efficacy of immunomodulating therapies in comparison to supportive care only related to mortality and for providing hypotheses on most promising treatment.

